# The Role of Flavonoids in Invasion Strategy of *Solidago canadensis* L.

**DOI:** 10.3390/plants10081748

**Published:** 2021-08-23

**Authors:** Artur Likhanov, Marian Oliinyk, Nataliia Pashkevych, Andrii Churilov, Mykola Kozyr

**Affiliations:** 1 Institute for Evolutionary Ecology NAS Ukraine, Akademika Lebedeva 37, 03143 Kyiv, Ukraine; MarianOlijnyk@gmail.com (M.O.); geobot2@ukr.net (M.K.); 2 Department of Botany, Dendrology and Forest Tree Breeding, The National University of Life and Environmental Sciences of Ukraine, Henerala Rodimtseva 19, 03041 Kyiv, Ukraine; churilov_konf@ukr.net; 3 M.G. Kholodny Institute of Botany of the NAS of Ukraine, Tereshchenkivska 2, 01601 Kyiv, Ukraine; pashkevych.nataly@gmail.com

**Keywords:** allelopathy, ammonia complexes, chemotaxis, flavonoids, growth regulator, PGPR, soil

## Abstract

This study provides data on the problem of potential complexation of phenolic compounds synthesized by the plants *Solidago canadensis* L. and *Solidago gigantea* Ait. with ammonium forms of nitrogen, partly immobilized in the soil. A comparative analysis of secondary metabolites of the studied plants was performed by HPLC. The leaves of invasively active *Solidago canadensis* contain nine times more rutin than the plants of *Solidago gigantea*. Adding to the leaf extracts (v/v1/20) aqueous ammonia solution to pH 8.0 on the chromatograms decreases the intensity or completely causes peaks of flavonoids to disappear; instead, there are peaks of new polar substances (tR 1.5 and 2.0 min). The selective effect of the phenol-ammonium complex on various plant species was revealed. At a concentration of 20 μg/mL, these substances stimulated the formation of lateral roots in soybean seedlings and chrysanthemum cuttings. The suppression of root growth in radish seedlings occurred at a concentration of flavonoids in the extract of 25 μg/mL. In addition, a positive chemotaxis of the *Pseudomonas putida* (PGPR) was detected in the nitrogen-containing complex based on rutin (5 μg/mL). The identified feature allows PGPR colonization of the root system of *Solidago canadensis* with corresponding changes in the structure of the microbial community. The ability of the obtained nitrogen-containing polar complexes to regulate the growth processes of plants at extremely low concentration points to promising research in this direction.

## 1. Introduction

The natural biogeographical process of migration of vascular plant species presupposes the presence of at least three conditions: The recipient habitat, the spreading species and the vector of transfer. At the same time, anthropogenic migrations of vascular plants differ, among others, in that they are caused by a significant transformation of one, two or all three components as a result of unintentional or purposeful human activity. Currently, the process of plant migration is accelerating, becoming global. The process of phytoinvasion is the fast distribution of alien plant species, accustomed to changing conditions, in new areas, sometimes intensively increasing the number [1]. In this regard, the study of physiological and biochemical mechanisms of the adaptation strategy of invasive plant species is extremely relevant in order to find out the signs and features of adaptations in the conditions of natural and anthropogenically altered environments that ensure the viability of populations [2]. Thus, the species *Solidago canadensis* L. (kenophyte of North American origin, epecophyte with the European-North American range, mesophyte, sciogeliophyte) and *S. gigantea* Ait. (kenophyte of North American origin with the European-American range, mesophyte, sciogeliophyte) deserve special attention [3,4,5,6]. 

*S. canadensis* is now actively spreading in the Forest and Forest-Steppe Zones of Ukraine. The species is a transformer of meadow vegetation and forest-fringe communities. It transforms plant communities, creating unbearable conditions of competition [4,7]. It quickly occupies the areas of disturbed phytocenoses through the vegetative propagation by long rhizomes and due to high growth rate, which in combination with the transformation of habitat leads to significant changes in the structure of local vegetation and has negative consequences for phytobiota and zoobiota [1,8,9]. Thus, this species is able to form monodominant communities, maintain its own populations for a long time, lead to collapse in the recovery process, i.e., significantly extend the recovery period of indigenous plant communities of meadow vegetation [10,11,12].

The reasons for the intensive expansion of this species, despite the close attention of scientists around the world, remain controversial [13,14,15,16]. Two provisions are the most discussed. One of those is based on the high allelopathic potency of plants [8,17,18]. The chemical composition of *S. canadensis*, in particular the compounds with allelopathic activity, is well studied [19,20,21,22]. The existing metabolic analysis has revealed 122 metabolites, including flavonoids, phenylpropanoids and terpenoids. The synthesis of substances is shown to significantly depend on the cytotype and ploidy of plants [23]. The invasiveness of polyploids is exceptional [24,25], and is primarily due to the increased production of biologically active metabolites [26]. The cytotype and ploidy are often overlooked in plant collection, which may explain the difference in quantitative levels of individual flavonoids and other metabolites in plant material [27]. In addition, plants are characterized by the daily dynamics of the content of phenolic compounds in the vegetative organs [28].

The complex action of metabolites when released into the soil can be accompanied by a significant change in the rhizosphere. The ability of secondary metabolites of *S. canadensis* to inhibit the growth of not only allelochemical-sensitive test cultures, but also highly stress-resistant weeds [29,30] has been experimentally confirmed. However, the complexity of this approach is that the study of allelopathic action of plant metabolites is mainly performed in artificial conditions, where to accurately reproduce the processes that occur in real natural conditions is extremely difficult. This is confirmed by reports that acid rainfall can significantly enhance the allelopathic effects of invasive species, in particular *S. canadensis*, on seed germination and growth of aboriginal species [31]. According to our observations, *S. canadensis* displaces other plant species selectively and with varying intensity. This may be due to the competitive relationship between plants for limited resources. In this regard, the ability of invasive species to compete fiercely for mineral nutrition, mainly nitrogen, is also widely discussed. It is indicated that invasive species actively assimilate ammonium forms of nitrogen. However, the problem is that soil colloids carry positive and negative electrical charges, with the latter being predominant in most soils [32]. This makes it possible to fix NH_4_^+^ cations by soil colloids in an exchangeable form and to protect them from leaching. The size of the soil particles can also influence the fixation of ammonium nitrogen. However, there is evidence that clay particles and silt are equally good at retaining NH_4_^+^ [32]. The preferential uptake of NH_4_+ over NO_3_^-^ despite the need for additional costs of ATP for pumping protons through PM occurs in plants under conditions of limited energy metabolism [33], for example, at low temperatures [34].

At the same time, aboriginal species usually do not show clear selectivity for the assimilation of certain forms of nitrogen. In addition, it has been shown that many invasive plant species are able to significantly slow down the activity of soil enzymes associated with the C, N and P metabolism [35,36]. This reduces the availability of nutrients in the soil. Such restrictions and imbalances in mineral nutrition may contribute to the rapid spread of invasive species, which are able to use forms of nutrients that are less accessible to other plant species. For example, in clay soils, which are preferred by *S. canadensis*, fixation of NH_4_^+^ increases with increasing pH and base saturation [32]. In soils with a high pH, the content of flavonoids secreted by the roots of *Solidago canadensis* prevails over the pool of common phenols. At the same time, in acidic soils, these plants predominantly release phenols and less flavonoids [37]. Flavonoids can form complexes with biogenic metal ions and other cations. Of practical interest are organic complexes, which in the presence of appropriate natural conditions, can be formed in plant tissues in the process of mineral nutrition. The barrier functions of secondary metabolites of plants are also important in terms of their potential ability to bind to metal ions when they enter plant tissues in subtoxic concentrations. In addition, ammonium forms of nitrogen are able to form organic complexes with flavonoids. The new complexes have different physico-chemical properties and, accordingly, other molecular targets in physiological processes. Hence, the life strategy of *S. canadensis* plants, in addition to the ability to secrete active metabolites and affect the mineral nutrition of other plant species, also includes the ability to impact the activity of soil enzymes produced by microorganisms [38,39,40].

Thus, the purpose of our studies was to conduct a comparative phytochemical analysis of plants *S. canadensis* and *S. gigantea*, to determine the most active secondary metabolites that are able to interact with ammonium groups and determine the specifics of their impact on plant growth and microorganism activity in the rhizosphere. 

## 2. Results

### 2.1. Comparative Analysis of the Secondary Metabolites in Leaves of Solidago Canadensis and Solidago Gigantea

**Figure 1:**
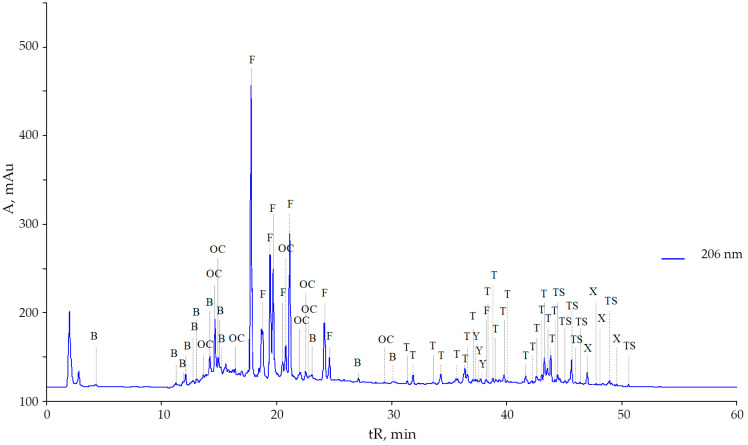
Chromatogram of methanolic extracts of *Solidago canadensis* L. leaves in the flowering phase: B—benzoic and oxybenzoic acids, OC—derivatives of oxycinnamic acids, F—flavonoids, T—terpenoids, TS—triterpenoid saponins, X—chlorophylls and their derivatives, Y—carotenoids and their derivatives.

More than 80 components, terpenoids, oxycinnamic and oxybenzoic acids, and 14 flavonoids (Figure 1) were identified by high-performance liquid chromatography (HPLC) in methanolic extracts of leaves and roots of *S. canadensis* and *S. gigantea*, collected in the park “Theofania” (Kyiv, Ukraine). In this work, we also relied on known studies of the biochemical composition of plants of the genus *Solidago* [41,42].

**Figure 2:**
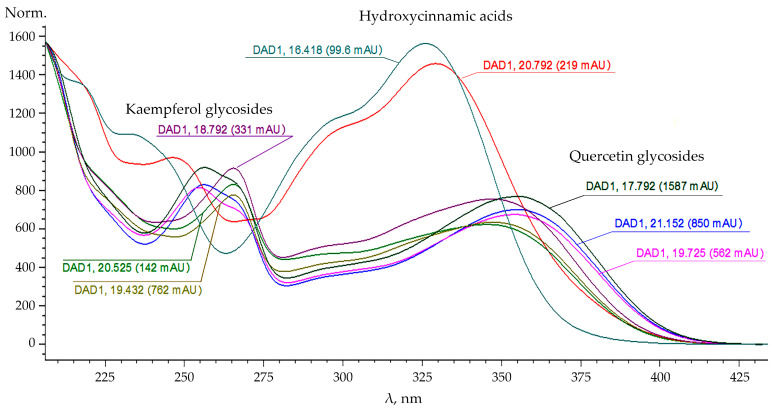
UV spectra of the main flavonoids of *Solidago canadensis* L.

UV spectra and retention times of the main peaks on the chromatogram in the presence of standards allowed identification of some flavonoids (Figure 2). Their main aglycones were kaempferol and quercetin. The largest share of the total pool of flavonoid glycosides in the leaves of the studied plants belonged to quercetin-3-*O*-beta-rutinoside (rutin).

**Figure 3:**
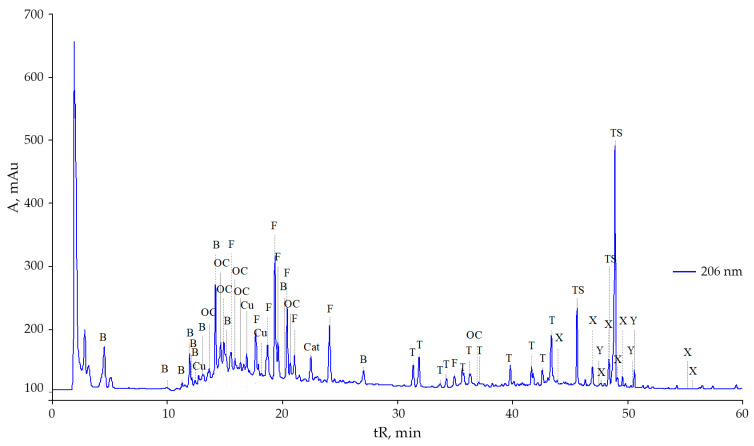
Chromatogram of methanolic extracts of *Solidago gigantea* Ait. leaves in the flowering phase: B—benzoic and oxybenzoic acids, OC—derivatives of oxycinnamic acids, Cu—coumarins, Cat—catechins, F—flavonoids, T—terpenoids, TS—triterpenoid saponins, X—chlorophylls and their derivatives, Y—carotenoids and their derivatives, ?— not identified.

The composition of the secondary metabolites of *S. gigantea* leaves has a very similar biochemical profile to *S. canadensis* (Figure 3). A very diverse composition of terpenoids and triterpenoid saponins was found in the leaves of *S. gigantea* plants in the flowering phase. Those compounds play an important role in the protective reactions of plants, regulation of water regime and interspecific relationships.

However, it is interesting that the two studied species have an almost identical composition of flavonoids, although their number is less in *S. gigantea* (Table 1). The content of rutin was 9 times, and isoquercitrin was 5.3 times lower in the leaves of *S. gigantea* than in those of *S. canadensis* according to the height of the peaks on the chromatograms (Figure 1 and Figure 3). However, leaves of *S. gigantea* showed a high content of presumably kaempferol-3-*O*-beta-rhamnoside (afselin). Also particularly noteworthy is the relatively high peak (tR 2.5 min) of a rather polar compound.

### 2.2. Modeling of Formation Processes of Organic Complexes of Flavonoids with Ammonium Nitrogen

Flavonoids are potentially capable of interacting with the ammonium forms of nitrogen. We simulated this process to study the ecophysiological properties of the generated phenol-ammonia complexes.

**Figure 4:**
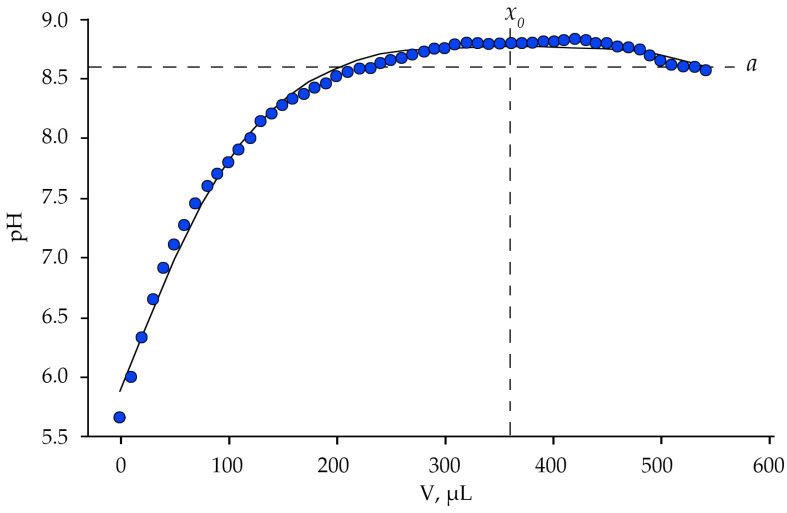
Lognormal model describing the dynamics of changes in the concentration of H^+^ ions in the aqueous extract of *Solidago canadensis* L. leaves; *x*_0_—the value corresponds to the volume of the titrant (NH_4_OH under appropriate conditions) at which the pH is stabilized, *a*—the calculated coefficient of the lognormal model corresponding to the pH value in a balanced system.

Equal volumes of aqueous ammonia solution (10%) were sequentially added to 100 mL of an aqueous extract of *S. canadensis* and *S. gigantea* leaves. When 10 μL of 10% aqueous NH_4_OH solution was added to the extracts, they acquired an intense dark brown color (Figure S1). The darkest solutions were at pH 8.0–8.2. Given that the aqueous solution of NH_4_OH is a weak base, in water it dissociates into NH_4_^+^ and OH^–^ ions. Under the conditions of binding of the ammonium group with 3-*O*-glycosides of flavonols, the balance of ions in the solution shifts towards the increase in OH^–^, but not as rapidly as in the case of dissolution of ammonia in water. At the time of maximum complexation, the leaf extract exhibits buffering properties, maintaining alkalinity at pH 8.75. As the titrant volume increases further, the equilibrium gradually shifts toward decreasing pH (Figure 4). 

The electrochemical content of the significant coefficients is related to the presence of metabolites in the extract, capable of complexation with NH_4_^+^ and a corresponding increase in the concentration of OH^–^ ions in the solution. The assumption that the NH_4_^+^ groups interact with flavonoids and organic acids with the formation of new complexes under the conditions of adding 10% aqueous solution of ammonia to the plant extract was confirmed by the results of chromatographic profiling.

**Figure 5:**
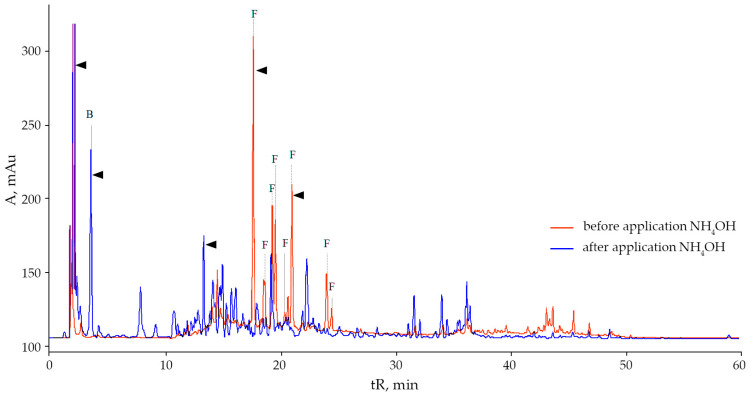
Chromatogram of aqueous extracts of *Solidago canadensis* L. leaves before (marked in red) and after (marked in blue) application of 10% NH_4_OH solution; F—flavonoids, B—benzoic and oxybenzoic acids.

On the chromatogram, the peaks corresponding to flavonoids completely disappeared or significantly decreased. Instead, two new peaks appeared at the beginning of the chromatogram with a fairly intense signal (Figure 5).

This indicates that the formation of new, rather polar compounds in the plant extract occurs mainly from mono- and diglycosides of flavonoids. An intense absorption peak with a retention time of 1.5 min was also found at the beginning of the chromatogram at a wavelength of 250 and 300 nm, and the next one is noted a little later (2.0 min) at a wavelength of 400 and 350 nm. Significant differences in the nature of light energy absorption by substances were detected in the flavonoids detected on chromatograms. For UV spectra, a hypsochromic shift for astragalin from 350 to 335 nm and a batachromic shift from 355 to 373 nm for isoquercitrin were established.

It was experimentally confirmed that under the presence of ammonium cations in plant tissues, the glucosides of kaempferol and quercetin are able to form polar nitrogen-containing complexes. Secondary metabolites of invasive species *S. gigantea* and *S. canadensis* are potentially able to regulate rooting processes. The leaf extracts of the studied species also include substances that can inhibit the development of the root system and growth processes in general. Potentially such compounds may include triterpenoid saponins, which are rich in leaves of the studied plants of the genus *Solidago*.

**Figure 6:**
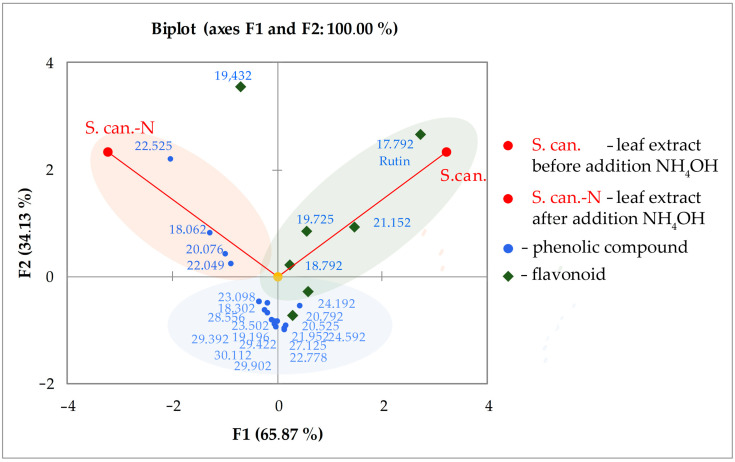
The result of the analysis of the chromatographic profiles of aqueous extracts of *Solidago canadensis* L. leaves by the method of analysis of principal components; numerical values correspond to the retention time of individual components in chromatography.

Principal component analysis confirmed that the biochemical composition of leaf extract is significantly altered by adding NH_4_OH. The axis of the first principal component (PC1) reflects the maximum data variance, which was mostly determined by flavonoids, mainly quercetin glycosides (indicated by diamonds and highlighted by an oval on the right along the PC1 axis) (Figure 6). 

The largest contribution to the total variance was made by rutin (tR = 17.792) and quercetin glycoside (tR = 21.152). The contribution of kaempferol glycosides (tR = 18.792, 24.192, and 20.525) to the variance was less significant. However, it should be noted that the kaempferol glycoside (presumably nicotiflorin) showed the greatest variance on the PC2 axis. This flavonoid, together with oxycinnamic acid (tR = 22.525), was found in the largest residual amount in the extract after treatment with ammonium. From the point of view of biological activity, three phenolic compounds (indicated by blue circles and highlighted by an oval on the left along the main axis, tR = 18.062, 20.076 and 22.049) are of particular interest. These were detected in the extract (S. can.-N) only after adding the corresponding volume NH_4_OH.

The proximity of individual substances on the coordinates of the axes of the principal components to the original composition of the extract (S. can.) indicates that they are contained in a relatively large amount and are easily transformed biochemically.

Comparative analysis of the biochemical composition of leaf extracts of two *Solidago* species and the results of PCA analysis made it possible to consider rutin as the most likely active substance. It was assumed that rutin in the root exudates of *S. canadensis* is able to react with ammonium cations of the soil and then, in the form of polar complexes, actively interact with native plant species and soil microorganisms.

When passing the extracts through the Al_2_O_3_ column, most flavonoids were retained or irreversibly bound to the sorbent (Figures S1–S2 ). The total content of kaempferol glycosides in leaf extracts was 2.0 times lower than quercetin. The ratio of the signal of the chromatograph detector at the peaks before and after adsorption decreased on average by 2.1–3.2 times (Table S1). It was found that Al_2_O_3_ has the highest adsorption capacity in relation to rutin and presumably afzelin. Their content in the extracts decreased more than 3 times. It was assumed that rutin in the root exudates of *S. canadensis* is able to react with ammonium cations of the soil and then, in the form of polar complexes, actively interact with native plant species and soil microorganisms.

### 2.3. Experimental Determination of Biological Activity of Flavonoids in Complex with Ammonium

**Figure 7:**
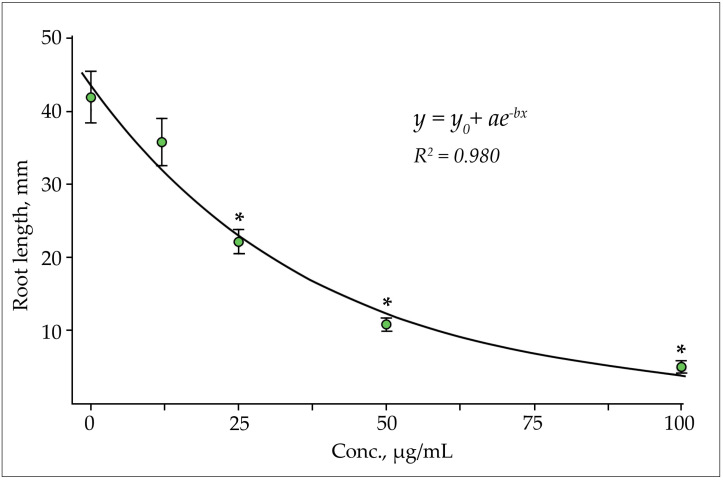
Inhibition of root growth of radish seedlings by phenol-ammonium complex depending on the content of flavonoids in the leaf extract of *Solidago canadensis* L.; the data were compared using Tukey’s test (HSD) by one-way ANOVA: *—significant differences to the control at the level *p* < 0.01.

The study of the effect of phenol-ammonium complexes revealed significant differences in their effect on the growth processes of different plant species. The polar complex obtained by adding an aqueous solution of ammonia to the extract inhibited the growth of roots of radish seedlings. The degree of growth inhibition (allelopathic effect) was strictly inversely correlated with the concentration of flavonoids (rs = −1.0, significance level α = 0.05). The most active effect on root growth was observed at a concentration of flavonoids in the leaf extract of 50–100 μg/mL (Figure 7). 

The leveling of the inhibitory effect depending on the concentration of active substances is fairly accurately approximated by an exponential function (*y* = 0.7166 + 42.9168e^−0.256^, *R*^2^ = 0.980). The significance of differences with the control (*p* < 0.01) was confirmed in the variants with the concentration of flavonoids in the initial extract exceeding 25 μg/mL.

**Figure 8:**
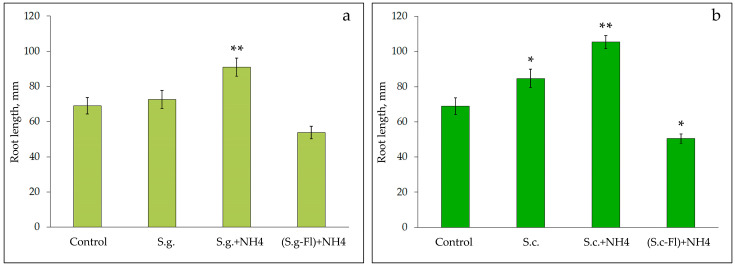
Influence of water extracts of *Solidago giganrea* (**a**) and *Solidago canadensis* (**b**) leaves on the growth of roots of *Glycine max* seedlings (n = 30): S.g., S.c.—initial extracts; S.g.+NH_4_, S.c.+NH_4_—extracts with added ammonia; (S.g.-Fl)+NH_4_, (S.c.-Fl)+NH_4_—extracts with added ammonia after adsorption in Al_2_O_3_ column; the data were compared using Tukey’s test (HSD) by one-way ANOVA: *—significant differences to the control at the level *p* < 0.05; **—at the level *p* < 0.01.

The opposite effect was found when growing soybean seeds and processing chrysanthemum shoots. A moderate stimulating effect on root growth was determined for the aqueous extract of *Solidago gigantea*, which increased significantly under the formation of phenol-ammonia complexes (Figure 8a). The difference with the control was significant only in the extracts with the addition of 10% ammonia solution (*p* < 0.01). In the extract of the leaves of this plant species with a reduced content of flavonoids, the stimulating effect was leveled and inhibition of growth processes was observed. A similar but more pronounced effect was found with the use of *S. canadensis* extracts (Figure 8b). Under conditions of partial extraction of flavonoids with aluminum oxide, the effect of the aqueous extract of leaves on the growth of soybean roots was significantly reduced (*p* < 0.01) compared to the original extract and its modification with ammonia solution.

**Figure 9:**
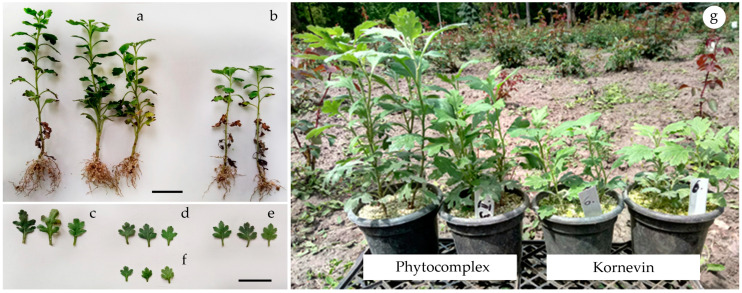
Stimulating effect of phenol-ammonia complexes on the growth and development of chrysanthemums: cuttings of the variety ‘Opal’: a—treatment with rutin-ammonia complex, b—‘Kornevin’preparation (control); leaves of chrysanthemum variety ‘Valeria’after a single treatment: c—rutin-ammonia complex, d—‘Kornevin’ (IBA), e—phytocomplex (extract of *Solidago canadensis* L. leaves with aqueous ammonia solution), f—water; g—plants of the variety ‘Queen of Autumn’ grown as container crops, after a single treatment with phytocomplex and ‘Kornevin’ (IBA).

An aqueous solution of the phenol-ammonia complex showed high efficiency when used as a root growth stimulator in grafting chrysanthemums. For three weeks of cultivation, the cuttings formed a fairly developed root system (Figure 9a).

The planting material of studied varieties obtained by using the phenol-ammonia complex was significantly larger in terms of morphometric parameters. The plants had longer shoots and a more developed root system (Figure 9g). Notably, cuttings treated with a phenol-ammonium complex obtained on the basis of rutin had an increase in the linear dimensions and leaf area (Figure 9c). The obtained water-soluble complexes, even at a single application, can have a positive effect not only on the development of the root system, but also directly or indirectly stimulate the development of aboveground vegetative mass. Their prolonged stimulating effect indicates sufficient stability in the plant body and the ability to be transported by transport systems in the meristematic tissues. At the same time, the stimulating effect of flavonoid-ammonium complexes was different for different varieties of chrysanthemums. However, during the formation of the final habitus, characteristic of the varieties, the difference in growth rate under the action of stimulants was leveled.

Thus, the assumption that flavonoids play a key role in stimulating growth processes was experimentally confirmed. The active formation of lateral roots in seedlings also deserves special attention. This fact indicates the high activity of pericycle cells. They are probably stimulated by the quite polar phenol-ammonia complexes, which with the flow of water through the apoplast, quite quickly get into the tissues that respond to the initiation of the formation of lateral roots. Stimulation of root system development is an important element of plant survival strategy in conditions of fierce competition for spatial resources and nutrients, as well as in conditions of moisture deficit. For adventitious species with a wide amplitude of adaptive reactions, the active formation of root systems is one of the main features that ensures the successful naturalization of plants in new conditions.

### 2.4. Positive Chemotaxis of Rhizobial Bacteria to the Rutin-Ammonium Complex

**Figure 10:**
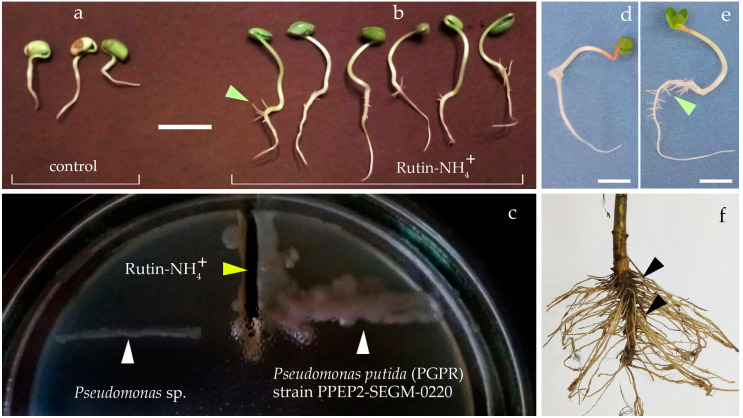
Effects of rutin-ammonia complex on seedlings and bacteria: a—control; b—stimulation of seed germination of test culture (*Glycine max* (L.) Merr.) with a solution of rutin-ammonium complex (20 μg/mL); c—positive chemotaxis of rhizobial bacteria *Pseudomonas putida* (PGPR) strain PPEP2-SEGM-0220 for 48 h; *Pseudomonas* sp.—bacteria that did not show chemotaxis; *Raphanus sativus* var *radicula* Pers. sprouts: d—control; e—after treatment (5 μg/mL); f—root of *Solidago canadensis;* b, e, f—arrows show active formation of lateral roots; scale bar: a, b—3 cm; d, e —1 cm.

Similar results were obtained when testing the newly formed complex of rutin with an aqueous solution of ammonia (Rutin—NH_4_^+^) on soybean seeds. The stimulation of root growth was observed at a concentration of 20 μg/mL. Increasing the concentration to 100 μg/mL inhibited seed germination and seedling formation (Figure 10). An essential feature of the action of the studied complex is its ability to stimulate the formation of lateral and adventive roots (Figure 10b,e). This specificity of root formation is characteristic of *S. canadensis* (Figure 10f). The actively forming lateral and adventive roots are located in the surface layer of the soil, which is the most populated by microorganisms. A significant surface area of young roots contributes to the active release of various metabolites into the soil, including phenolic substances. 

Rhizobial bacteria *Pseudomonas putida strain* PPEP2-SEGM-0220, which we isolated from soybean seed skin and identified by 16S RNA, have growth-stimulating activity in relation to other representatives of legumes. In the presence of the Rutin–NH_4_+ complex (5 μg/mL) in the nutrient medium, rhizobial bacteria showed positive chemotaxis (Figure 10c). In addition, the presence of this compound contributed to the active growth of the colony. At the same time, other bacteria of the genus *Pseudomonas* did not show explicit chemotaxis.

## 3. Discussion

In the plant body, phenolic compounds affect the hormonal regulation of morphogenesis [45,46] and perform a wide range of regulatory and protective functions [47,48,49,50]. Considering the invasive strategy of plant species, the biotransformation and complexation of secondary metabolites is especially interesting in the rhizosphere under conditions of their entry into the soil. It is known that under conditions of limited nitrogen nutrition in plant tissues, the content of total phenols and flavonoids increases [51]. Since the vast majority of phenylpropanoids and flavonoids are capable of complexation with cations, phenolic compounds can be transformed when they enter the soil. Thus, quercetin aglycones and glycosides actively interact with ammonium groups to form highly polar water-soluble compounds. Additionally, phenol-ammonia complexes are easily soluble in water and become available to the roots. This is important because the availability of nitrogen limits the nutrition of the vast majority of plants [52,53]. As we have shown, the newly created nitrogen-containing complexes can stimulate the growth of lateral roots and increase the total effective area of the root system. Flavonoids are actively secreted by the root system of *Solidago canadensis*. They are extremely reactive towards ammonia complexes. Polar compounds formed as a result of a chemical reaction are biologically active. Their regulating effect on growth processes is observed even at concentrations comparable to those of phytohormones. 

However, an increase in the concentration of phenol-ammonia complexes by 3–5 times has a noticeable effect of suppressing the growth of test cultures, which can also be considered as an allelopathic effect. A similar effect was confirmed for kaempferol-3-*O*-β-D-glucoside isolated from the roots of *Solidago canadensis*. An increase in its concentration from 15 µg/mL and higher enhanced the suppression of shoot growth of *Echinochloa colona* (L.) Link. [54]. 

It is believed that different plant species differ in their ability to absorb various forms of nitrogen [55]. Invasive species are quite competitive for macro- and micronutrient nutrition in the soil, which in turn leads to the suppression of local flora [56]. Compared to aboriginal species, many invasive plants have the ability to effectively absorb nitrogen [57]. Thus, *Flaveria* *bidentis* (*L*.) *Kuntze*, a species invasive for northern China, significantly increases bioproductivity in response to increasing nitrogen content. In addition, the invasive species, compared to local species *Amaranthus retroflexus* L. and *Eclipta prostrata* (L.)L., showed a significant advantage over ammonium forms of nitrogen, while local species did not show a clear preference for certain forms of nitrogen [55].

According to the “novel weapons hypothesis”, the root exudates of migratory species can severely suppress aboriginal plants, due to their maladaptation or lack of available nutrients. In this case, it is convincing to view the advantages of new competitive features as an alternative to the compromise of “growing or defending”, which underlies the theory of evolution of increased competitiveness [58].

We believe the strategy of *S. canadensis* to be in the development of spatial and other vital resources as a set of features, which is due to the ability of plants to extensively produce secondary metabolites, which are actively deposited in the rhizosphere. Since the intensity of their synthesis directly depends on the ploidy of plants, it is expected that the greatest transformation of the environment will be due to the increase in populations of polyploid cytotypes. It is also important that *Solidago canadensis* plants under the conditions of introduction significantly enhance the synthesis of secondary metabolites with high allelopathic potential, which confirms their direct relationship with plant invasiveness [59].

An important condition for the successful invasion of alien invasive species is the formation of their positive feedback with soil microorganisms [60,61]. Recent studies have shown that content of quercetin is high in root exudates of invasive plants *Triadica sebifera* (L.) Small. The substance, as a part of exudates, is key in signaling for the interaction of plants with mycorrhizal fungi and soil microorganisms in general [62].

Mostly through the root secretions, *Solidago canadensis* plants affect the enzymatic activity of microorganisms, which in stable conditions usually provide the availability of nutrients for other plant species. The potential ability of phenolic compounds to interact with ammonium cations immobilized on the surface of soil particles and the formation of highly polar compounds, creates specific conditions for nitrogen nutrition of plants. This feature of the plant *Solidago canadensis* gives it an extraordinary advantage over other species. In addition, new polar complexes diffuse rapidly in soil solutions, are bioavailable, and at high concentrations (above 25 μg/mL) significantly inhibit the growth of root systems of competing plants.

**Figure 11:**
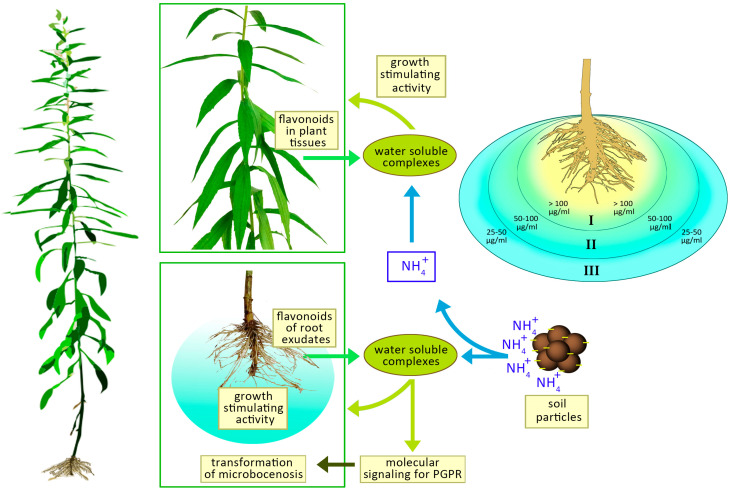
General scheme of the process of formation of water-soluble compounds in the case of interaction of flavonoids of *Solidago canadensis* L. with ammonia complexes that enter through the roots into plant tissues or are formed directly in the soil due to the active release of flavonoids by the root system.

Based on the experimental data we have obtained, three zones can presumably form around the root system of the *Solidago canadensis*: I—zone of high allelopathic activity (the content of flavonoids is above 100 μg/mL), II—zone of medium activity (50–100 μg/mL) and III—zone of low activity (25–50 μg/mL). In the zone I, the germination of seeds and the development of seedlings can be suppressed, in the zone II—inhibition of the growth of many plant species, in the zone III—inhibition of the growth of susceptible plant species. The spatial position and size of the designated zones are probably largely determined by the composition of the soil, the amount of moisture, the content of ammonium forms of nitrogen, and the intensity of biologically active substances released by plants (Figure 11). 

Experimental confirmation that phenol-ammonia complexes cause positive chemotaxis in some species of rhizobial bacteria confirms that the plants are able to selectively change the composition of microorganisms of the rhizosphere and rhizoplan.

In the study of the influence of a complex of microorganisms on the nutrition of *Solidago canadensis* plants, it was shown that plants are positively affected by the higher species diversity of bacteria. It is also interesting to note that inoculation of the soil of model plants *Solidago canadensis* with microorganisms isolated in the zone of invasive distribution of this species in southern China caused a significant increase in plant bioproductivity, which enhances their competitive dominance. The complex of microorganisms from the pastures of Inner Mongolia, where this species has not yet spread, did not cause such an effect.

At the same time, a synergistic effect of a mixed complex of microorganisms on the formation of aboveground mass of plants was determined, which was much better in the presence of sufficient nutrients and moisture [63]. Moisture is a necessary condition for the complexation and transport of nutrients in the soil. Absorbed through the roots, phenol-ammonia complexes move acropetally along the transport system of xylem, as well as laterally through the parenchyma of xylem and bark. Low concentrations of these compounds initiate the formation of lateral roots from the pericycle. It is important to note that the compounds thus obtained are unstable, and in the process of deep oxidation can be converted into other nitrogen-containing organic substances that actively interact with phosphate ions and also form extremely active substances, the chemical structure of which has yet to be determined.

## 4. Materials and Methods

### 4.1. Collection and Phytochemical Studies of Plant Material

#### 4.1.1. Source Plant Material

Plant material (ramets) of *Solidago gigantea* and *Solidago canadensis* was collected during the flowering phase in August-September 2018–2020. All ramets for analysis were obtained within the ruderal habitats of sand deposits in the vicinity of park “Theofania” in Kyiv, Ukraine (I 2.242 Ruderal biotopes of sand deposits, EUNIS I1.5 Bare tilled, fallow or recently abandoned arable land). Habitat types are listed according to the biotope classification for the Forest and Forest-Steppe Zones of Ukraine [64]. In the studied habitats, plant communities dominated by *Solidago gigantea* and *Solidago canadensis* form the association *Rudbeckio laciniatae-Solidaginetum canadensis* (Tüxen et Raabe ex Anioł-Kwiatkowska 1974). Geographic coordinates of collection site: 50°20′21.0″ N 30°30′03.1″ E and 50°20′16.0″ N 30°30′47.2″ E.

Leaf extraction was performed with methanol in the ratio of dry mass (DM) to alcohol: *v*/*v*—1/10. The aqueous extract was obtained from the leaves of *Solidago canadensis* and *Solidago gigantea* with double-distilled water (*v*/*v*—1/20). A portion of dry leaves was filled with hot water (T = +80 °C) and kept for 2 h in a water bath. The extract was filtered and stored at T = +4 °C.

#### 4.1.2. Methods of High-Performance Liquid Chromatography

The leaves after drying were thoroughly ground and extracted with methanol at ratio of 100 mg of dry weight per 1 mL, respectively, for a day, in a room protected from light, at room temperature. The material in this state until chromatographic separation was stored in a freezer at −15 … 20 °C, and immediately before analysis was filtered through a syringe filter 0.2 … 0.5 µm. Samples were separated using reversed-phase high performance liquid chromatography (HPLC) on an Agilent 1100 system, according to the 2-eluent scheme (eluent I = 0.05 M aqueous solution of orthophosphoric acid; eluent II = acetonitrile) on a column of Thermo Scientific Hypersil ™ BDS C18. Sample volume 5 μL, column temperature 20 °C and after 45 min 40 °C, flow rate 0.3 mL/min and after 30 min −0.6 mL/min; analysis time up to 80 min, elution profile—isocratically 1% eluent II in eluent I for 2 min, then a linear gradient from 1% to 99% II in I for 30 min, and finally, isocrat 99% II in I for 20 min and more. Detection at wavelengths of 206, 254, 300, 350 and 450 nm to determine most organic compounds (including terpenoids), most substances of aromatic nature, phenylpropanoids (oxycinnamic acids and lignans), flavonoids (flavones and flavonols), carotenoids and chlorophylls, respectively. Table 2 shows the values that were used to assign signals to the chromatograph.

Absorption spectra in the ultraviolet and visible ranges were recorded for all substances in order to establish the nature of the secondary metabolites and to assign the chromatographic peaks to certain groups of substances. Standards of chlorogenic acid and rutin were used to verify retention time ranges, spectra, and approximate estimates. The β-sitosterol standard was used as a lipophilic component with low molar extinction. 

### 4.2. Preparation of Phenol-Ammonium Complexes from Plant Extracts and Flavonoids

#### 4.2.1. Obtaining Complexes Based on Aqueous Extracts of Leaves with a Solution of Ammonia 

To obtain phenol-ammonia complexes of secondary metabolites, 10 μL of an aqueous solution of ammonia (10%) was gradually added to 100 mL of aqueous extracts of leaves of *Solidago canadensis* and *Solidago gigantea* (*v*/*v*—1/20) to a pH of 8.0. During the addition of NH_4_OH, the extracts acquired an intense dark brown color. The darkest solutions were at pH 8.0–8.2. The dependence of pH value on the volume of NH_4_OH is quite accurately described (*R*^2^ = 0.9922) by the modified Gaussian Equation (1):

(1) $$y=ae^{\left[-0.5(\frac{\left|x-x_{0}\right|}{b}{)}^{c}\right]}$$

The coefficients of the regression equation are presented in Table 3.

In outlining the chemical processes, the value of *x*_0_ corresponds to the maximum volume of titrant (NH_4_OH under appropriate conditions), at the introduction of which the pH is stabilized. The coefficient *a* corresponds to the pH value, which is balanced in the system before and after titration. The coefficient *c* determines the steepness of the function (pH value) under the conditions of addition to the extract of NH_4_^+^ and OH^–^ ions. A decrease in *c* value in the equation may indicate the presence in the extract of a significant amount of organic acids and phenols capable of reacting with the formation of salts, nitrogen-containing organic compounds and water. At the same time, the buffering properties of the extract will decrease. The coefficient *b* in the equation has the opposite meaning. Its increase may indicate a relatively low pool of organic acids in the aqueous extract, but a much higher content of phenolic compounds, in particular flavonol glycosides, which are able to form complexes with the ammonium group. 

#### 4.2.2. Obtaining Polar Complexes Based on Rutin with an Aqueous Solution of Ammonia

A total of 10 mg of rutin (Merck, Darmstadt, Germany) was vortexed in 300 μL of double-distilled water for 60 s in a test tube. Then 100 μL of 10% aqueous ammonia solution was added and stirred again for 30–40 s until complete dissolution of the contents of the tube. The resulting solution was dark brown. The volume of the solution was adjusted with double-distilled water to 1 mL and stored at +4 °C. The resulting stock solution contained 10 mg/mL of polar substances.

#### 4.2.3. Adsorption of Secondary Metabolites from Leaf Extracts of *Solidago Canadensis* and *Solidago Gigantea* on Al_2_O_3_ and Determination of Flavonoid Content

To determine the most active (against the test cultures) substances contained in the leaves of the test plants, 200 mg of Al_2_O_3_ powder (Merck, Darmstadt, Germany) for chromatography was added to 2 mL tubes (n = 4) to 1 mL of extract. The contents of the tube were vortexed thoroughly for 1 min and allowed to stand for 5 min at room temperature. The procedure was repeated 3 times. Next, these tubes were centrifuged for 5 min at 6000 rpm. The supernatant was carefully collected without the sorbent, transferred to new tubes and used for testing.

The quantitative content of flavonoids in leaf extracts before and after adsorption on Al_2_O_3_ was determined by spectrophotometry (Optizen Pop, Mecasys, *Daejeon*, South Korea) at λ = 419 nm. 200 μL of 0.1 M solution of aluminum chloride (AlCl_3_) and 300 μL of 1 M sodium acetate (CH_3_COONa) were added to 300 μL of extract. The calibration graph was based on quercetin (Sigma-Aldrich, Darmstadt, Germany). The phytochemical experiments were repeated 4 times.

### 4.3. Bioassay

#### 4.3.1. Germination of Seeds of Test Crops and Conditions for Processing and Growing Plants

Seeds of *Raphanus sativus* var *radicula* Pers of the variety ‘Rozovo-krasniy s belim konchikom’ (bred in 1940 by the All-Russian Research Institute of Vegetable Breeding and Seed Production with the participation of the Biryuchekutskaya Vegetable Breeding Experimental Station) and soybeans *(Glycine max* (L.) Merr.) of the variety ‘OAC Strive’ (Canada) were used to determine the biological activity of *Solidago canadensis* and *Solidago gigantea* leaf extracts. A total of 30 and 20 seeds of test cultures, respectively, were germinated in Petri dishes on wet filter paper in a thermostat at 25 °C (n = 4). The effect of extracts based on ammonium complexes on growth processes of radish and soybean was assessed on length of seedlings roots on day 5.

To study the effect of phenol-ammonia complexes of *Solidago canadensis* leaf extract on the processes of rhizogenesis, three varieties of chrysanthemums *(Chrysanthemum x koreanum Makai)* ‘Opal’, ‘Valeria’ and ‘Queen of Autumn’ from the collection of the Institute of Evolutionary Ecology of the NAS of Ukraine were used. The lower ends of freshly cut chrysanthemum shoots (n = 30) with 4–5 nodes were immersed for 6 h in water, to which was added the resulting complex (*v*/*v*–1/20). The control batch of shoots (n = 30) was immersed in water for 5 h 40 min. Then the control shoots were soaked for 20 min in a solution of ‘Kornevin’, which was prepared fresh, according to the manufacturer’s recommendations. The active substance in this preparation is indolyl butyric acid (IBA), a well-known phytohormone of the auxin class. An amount of 1 L of prepared solution for rooting of shoots contains 5 mg of IBA. After soaking, the experimental and control batches of shoots were transferred into containers with perlite for 21 days for rooting. Next, the cuttings were transplanted into containers with a soil mixture based on chernozem-peat-perlite (*v*/*v*/*v*–1/1/1) and grown for 1 month.

#### 4.3.2. Study of Chemotaxis of *Pseudomonas* Sp. Bacteria

To study the chemotaxis of bacteria in relation to the complex obtained by the interaction of rutin with an aqueous solution of ammonia, we used the previously isolated strain *Pseudomonas putida* PPEP2-SEGM-0220 (GenBank: MW255059.1), which by a number of indicators belongs to the group PGPR (plant growth promoting rhizobacteria); *Pseudomonas* sp. isolate, which did not show the ability to stimulate plant growth and development, served as a control. A shallow narrow groove was made in King B Medium on Petri dishes to introduce phenol-ammonia complex at a concentration of 5 µg/mL. Bacterial cultures were applied in a dash across the groove. Petri dishes were kept in a thermostat for 28 °C for 2 days. The nature of growth and the distance of bacterial colonies to the complex introduced into the groove were determined.

### 4.4. Methods of Digital and Statistical Data Processing

Photodocumentation and digital image processing were performed in a specialized program Image-Pro Premier 9.0 (Media Cybernetics, Rockville, MD, USA). The significance of the differences between the values (*p* < 0.05) was determined by the analysis of variance (ANOVA) method in the XLSTAT (Addinsoft Inc., New York, NY, USA, 2010). The data were compared using Tukey’s test. The principal components analysis (PCA) was performed in the XLSTAT program. SigmaPlot 12.0 (Systat Software Inc., San Jose, CA, USA, 2011) was used for regression analysis.

## 5. Conclusions

The analysis of flavonoids in extracts of the invasive species *Solidago canadensis* revealed a significant increase in the content of rutin (quercetin-3-*O*-beta-rutinoside) in its composition compared to *Solidago gigantea*. When entering the rhizosphere with exudates, flavonoids (mainly quercetin glycosides) can interact with ammonium cation, either free or immobilized on negatively charged surfaces of soil particles. As a result, new polar compounds are formed. If there is enough water in the soil, they are able to actively diffuse. The effect of the phenol-ammonia complex on plants of different families (*Asteraceae* Bercht. & J. Presl, *Fabaceae* Lindl., *Brassicaceae* Burnett) differs not only in the strength of the effect, but also in the influence (stimulation or suppression). At low concentrations (20 μg/mL), these substances stimulate the formation of lateral and adventitious roots in soy-bean and chrysanthemum plants, and in concentrations more than 100 μg/mL) inhibit them. Radish sprouts are more susceptible to aqueous extracts of *Solidago canadensis* leaves. The suppression of the growth of their roots is significant at a concentration of flavonoids in the extract of 25 μg/mL. One of the possible explanations for the selective action of the phenol-ammonia complex may be the similarity or difference of metabolic pathways associated with growth-regulation mechanisms in the studied plants. However, to prove this assumption, it is necessary to carry out special studies on a larger number of species from these families. 

Rutin, after interacting with ammonium in the form of a new complex, easily dissolves in water and causes positive chemotaxis in symbiotic bacteria of the PGPR group (plant growth promoting rhizobacteria), which stimulate the formation of the root system and enter into competitive relationships with phytopathogenic microorganisms. The formation of a developed system of lateral and adventitious roots contributes to the use of the most nutrient-rich surface layers of the soil inhabited by microorganisms. Enhanced synthesis and release of flavonoids, therefore, is an important element of the strategy for the development and biotransformation of a new habitat.

## Figures and Tables

**Table 1:** Comparative evaluation of the composition of the main group of flavonoids in the leaves of *S. canadensis* and *S. gigantea*.

Flavonoid	*S. canadensis*		*S. gigantea*		Absorption Peak, nm	Ref.
	Retention Time, min	mAU	Retention Time, min	mAU		
Rutin (quercetin-3-*O*-beta-rutinoside)	17.762	1587	17.674	176	256, 355	[43]
Astragalin (kaempferol-3-*O*-beta-glucoside)	18.792	331	18.714	106	266, 350	[43]
Nicotiflorin (kaempferol-3-*O*-beta-rutinoside)	19.432	762	19.354	402	266, 347	[44]
Isoquercitrin (quercetin glycosid)	19.725	562	19.621	107	255, 355	[42]
Afzelin (kaempferol-3-*O*-beta-rhamnoside)	20.525	142	20.407	249	266, 347	-
Quercetin glycoside 1	21.152	850	21.048	70.8	256, 356	-
Kaemperfol glycoside	24.192	323	-	-	265, 347	-
Quercetin glycoside 2	24.592	142	-	-	254, 355	-

**Table 2:** Symbols of peaks on the chromatogram.

Compounds	Symbols
Derivatives of phenols, benzoic and oxybenzoic acids	B
Catechins	Cat
Coumarins	Cu
Derivatives of oxycinnamic acids	OC
Flavonoids	F
Sterines	S
Terpenoids	T
Triterpenoid saponins	TS
Chlorophylls and their derivatives	X
Carotenoids and their derivatives	Y

**Table 3:** Coefficients of the modified Gaussian function describing the process of complex formation.

Coefficient and Its Value		Standard Error	t	*p*
*a*	8.7497	0.0138	635.8646	<0.0001
*b*	390.5112	6.4907	60.1646	<0.0001
*c*	3.9553	0.1341	29.4951	<0.0001
*x*_0_	369.2351	6.0684	60.8459	<0.0001
*R* ^2^	0.9922	-		

## Data Availability

The data presented in this study are available on request from the corresponding author.

## Supplementary Materials

The following are available online at https://www.mdpi.com/article/10.3390/plants10081748/s1, Figure S1: Color of aqueous extracts of leaves *S. gigantea* and *S. canadensis*, with water (H_2_O) and 10% NH_4_OH solution; dilution of extracts—*v*/*v*–1/1; Figure S2: Chromatogram of aqueous extracts of *S. canadensis* leaves before adsorption on Al_2_O_3_; Figure S3: Chromatogram of aqueous extracts of *S. canadensis* leaves after adsorption on Al_2_O_3_; Table S1: Adsorption capacity of Al_2_O_3_ for flavonoids from aqueous extract *S. canadensis*.
