# Supplementary material for: The Role of Flavonoids in Invasion Strategy of Solidago canadensis L
Source: Plants (Basel). 2021 Aug 23;10(8):1748. doi: 10.3390/plants10081748 (PMC8401302; doi:10.3390/plants10081748)
Supplement: Supplementary file 1 [file plants-10-01748-s001.zip › plants-1337123-supplementary.pdf]

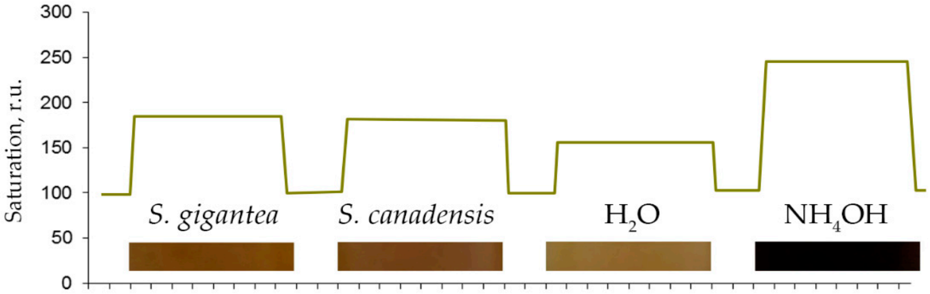

Figure S1. Color of aqueous extracts of leaves *S. gigantea* and *S. canadensis*, with water (H_2O) and 10% NH_4OH solution; dilution of extracts: v/v – 1/1

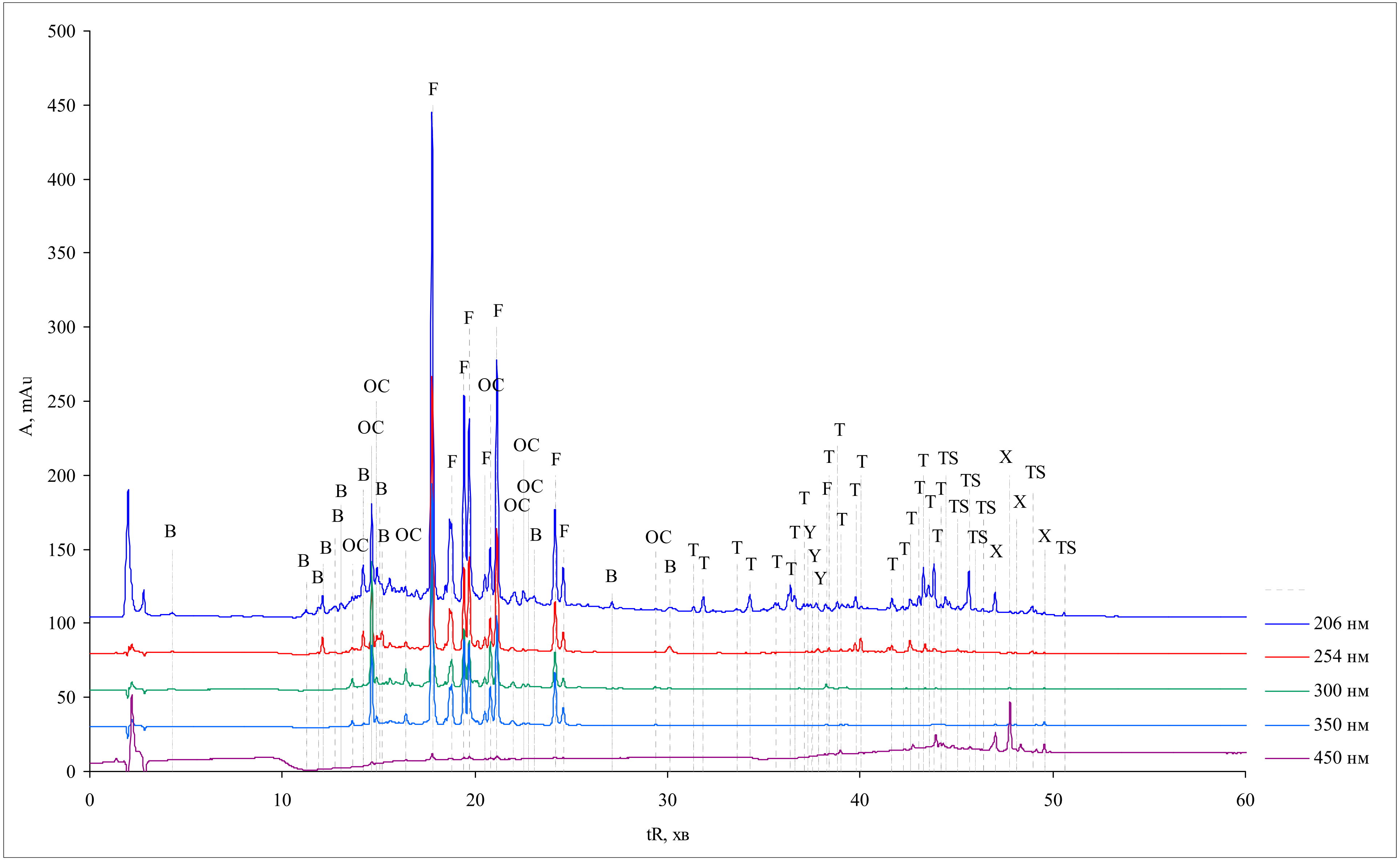

Figure S2. Chromatogram of aqueous extracts of *S. canadensis* leaves before adsorption on Al_2O_3

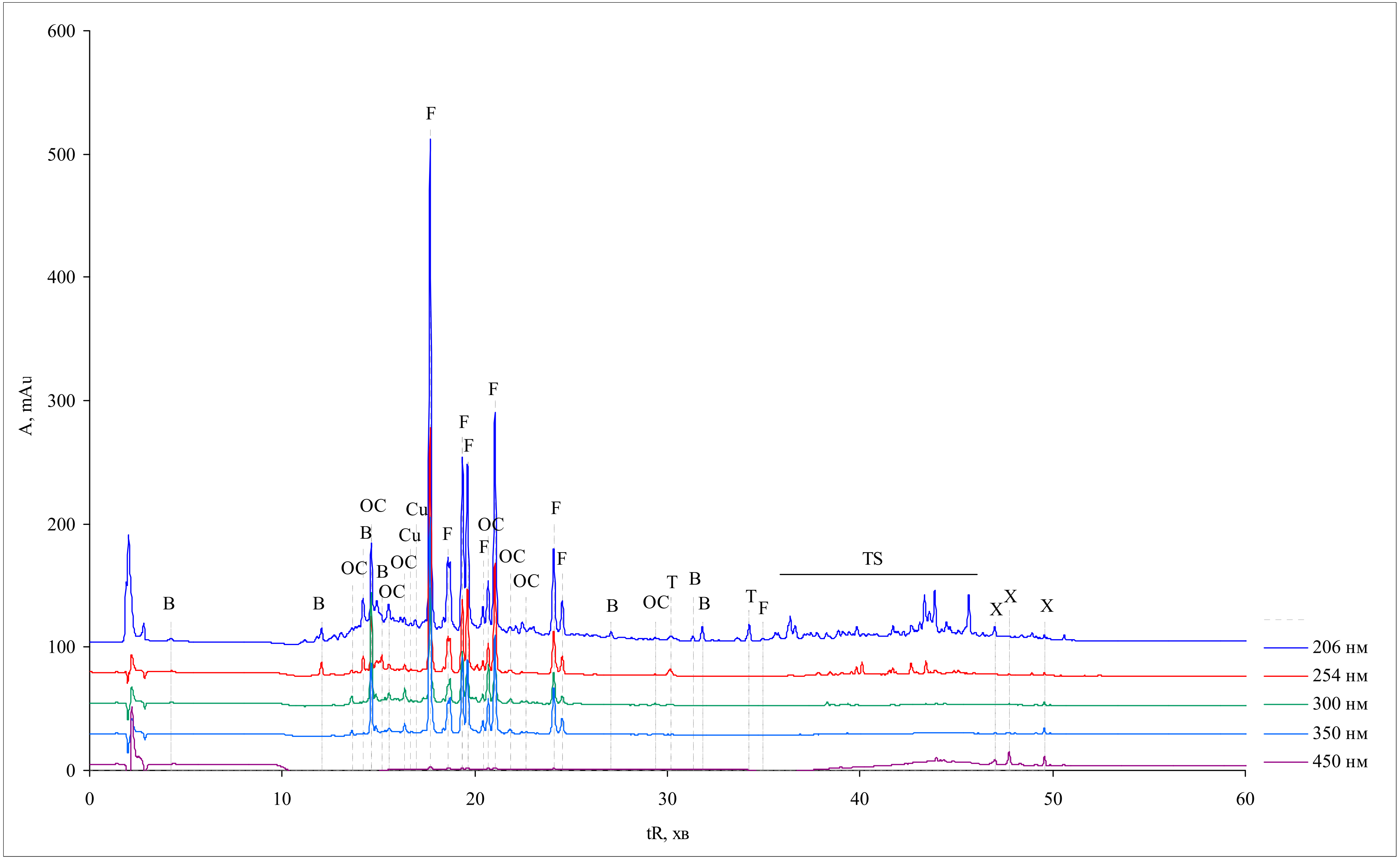

Figure S3. Chromatogram of aqueous extracts of *S. canadensis* leaves after adsorption on Al_2O_3

Table S1. Adsorption capacity of Al_2O_3 for flavonoids from aqueous extract *S. canadensis*

Flavonoid	mAU		Ratio
	**before Al_2O_3**	**after Al_2O_3**	
Rutin quercetin-3-O-beta-rutinoside	1587	517	**3,07**
Astragalin kaempferol-3-O-beta-glucoside	331	117	**2,83**
Nicotiflorin kaempferol-3-O-beta-rutinoside	762	287	2,66
Quercetin glycoside (Isoquercitrin)	562	253	2,22
Afzelin camperfol-3-O-beta-rhamnoside	142	45	**3,16**
Quercetin glycoside 1	850	361	2,35
Kaemperfol glycoside	323	157	2,06
Quercetin glycoside 2	142	59	2,41
Total camperfol glycosides	1558	606	2,57
Total quercetin glycosides	3141	1190	2,64
**The amount of flavonoids**	4699	1796	**2,62**
